# Retraction: “Niches of nine mangrove species in a *Sonneratia apetala*‐colonized area of Dongzhai Harbor, Hainan Island, China” by Wu, F; Liao, B; Chen, Y; Jiang, Z; Guo, Y; and Mei, L.

**DOI:** 10.1002/ece3.8511

**Published:** 2022-01-14

The above article from Ecology and Evolution, published online on 4 October 2020 in Wiley Online Library (wileyonlinelibrary.com), has been retracted by the journal Editors‐in‐Chief (Allen Moore; Andrew Beckerman; Jennifer Firn; Chris Foote; Gareth Jenkins; and Zoe Ma) and John Wiley & Sons Ltd. The retraction has been agreed due to major overlap with a previously published article written by many of the same authors.

## References

[ece38494-bib-0001] Wu, F. , Liao, B. , Chen, Y. , Jiang, Z. , Guo, Y. , & Mei, L. (2020). Niches of nine mangrove species in a *Sonneratia apetala*‐colonized area of Dongzhai Harbor, Hainan Island, China. Ecology and Evolution, 10, 11838–11846. 10.1002/ece3.6823 33145004 PMC7593169

